# Effect of Stored Humidity and Initial Moisture Content on the Qualities and Mycotoxin Levels of Maize Germ and Its Processing Products

**DOI:** 10.3390/toxins12090535

**Published:** 2020-08-20

**Authors:** Yun-qi Wen, Li-li Xu, Chang-hu Xue, Xiao-ming Jiang

**Affiliations:** College of Food Science and Engineering, Ocean University of China, No.5, Yushan Road, Qingdao, Shandong Province 266003, China; wenyq0715@163.com (Y.-q.W.); xulili8339@163.com (L.-l.X.); oucxuech@163.com (C.-h.X.)

**Keywords:** maize germ, zearalenone, deoxynivalenol, storage, maize germ oil, maize germ meal

## Abstract

With high fat and protein content, maize germ is easily infected with fungus and mycotoxins during its storage. The qualities and safety of germ and its processing products may be affected by the storage. However, studies on the effect of storage on quality and polluted mycotoxin level of maize germ are limited. In this study, maize germ was stored with different initial moisture contents (5.03, 9.07, 11.82 and 17.97%) or at different relative humidity (75, 85 and 95%) for 30 days. The quality indices of germ (moisture content and crude fat content) and their produced germ oils (color, acid value and peroxide value) as well as the zearalenone (ZEN) and deoxynivalenol (DON) levels of germ, oils and meals were analyzed. Results showed that maize germ with high initial moisture contents (11.82, 17.97%) or kept at high humidity (95%) became badly moldy at the end of storage. Meanwhile, the qualities of these germ and oils showed great changes. However, the ZEN and DON contents of this maize germ, oils and meals stayed at similar levels (*p* < 0.05). Therefore, the storage could produce influence on the qualities of germ and oils, but showed limited effect on the DON and ZEN levels of germ and their processing products. According to this study, the storage condition of germ with no more than 9% moisture content and no higher than 75% humidity was recommended. This study would be benefit for the control of germ qualities and safety during its storage.

## 1. Introduction

Consisting of endosperm and germ, corn is a main economic crop in the world [1]. At the same time, maize germ is a corn-processing byproduct, with high fat content (35–47%). It is also rich in various nutritional compositions, such as vitamin E and polyunsaturated fatty acids [2]. Maize germ is a good source of edible oil which is highly consumed around the world for its unique flavor [3]. Nowadays, large quantities of corn are used for producing alcohol and fuel alcohol, resulting in the production of large quantities of maize germ [4]. Maize germ is also used as an initial raw material to feed livestock such as swine, which are susceptible to the toxic effect of mycotoxins [5,6]. However, because of the high fat and protein content of maize germ, maize germ is more easily attacked by fungus and polluted with mycotoxins than corn and wheat, etc.—especially under the condition of appropriate temperature and humidity [7]. The growth and metabolism of mold not only leads to the bad organoleptic qualities of corn and germ, but also to the production of a range of mycotoxins, such as zearalenone (ZEN) and deoxynivalenol (DON).

**Figure 1:**
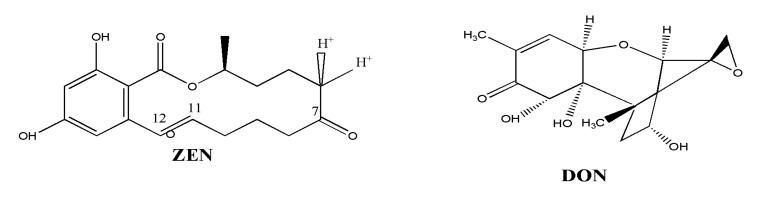
Molecular structures of zearalenone (ZEN) and deoxynivalenol (DON).

Zearalenone (ZEN) is usually produced by *Fusarium* species, including *Fusarium graminearum*, *Fusarium culmorum*, *Fusarium cerealis* and *Fusarium equiseti*, while DON can be produced by *Fusarium graminearum* and *Fusarium culmorum*. The molecular structures of ZEN and DON are shown in Figure 1. They are both considered as the most frequently detected mycotoxins in cereal and its byproducts, and they can pose heavy health risks for human beings as well as animals [8,9,10]. ZEN has been demonstrated to be estrogenic, hepatotoxic, immunotoxic and carcinogenic. DON has been shown to be acute or chronically poisonousness and exhibits carcinogenic, teratogenic and mutagenic characters [3,11]. Therefore, maximum permissible levels of ZEN and DON in foods have been established by many countries and organizations. The level of ZEN in corn oil must not exceed 400 μg/kg; levels of DON content in cereals intended for human direct consumption, cereal flour, bran and germ must not exceed 750 μg/kg [12].

Several food samples—including fiber-enriched bread, bran-enriched bread, cornflakes, popcorn and oatmeal—collected from Belgian supermarkets during April 2010 to October 2011 were detected to contain mycotoxins. The results show that all of these foods contaminated with an average of two to six mycotoxins. Maize and its byproducts were mostly infected with DON, ZEN and derivatives [13]. Another study reports that corn and its six derived milling fractions, including germ, bran, large and small grits, flour and animal feed flour, are all contaminated by mycotoxins. In particular, germ, bran and animal feed flour show marked concentration factors [14]. In terms of the distribution of DON in whole corn and milled corn fractions, endosperm, germ and pericarp contains 20, 25 and 55% of the original DON of kernel, respectively [15]. Meanwhile, ZEN is reported to be evenly distributed during the milling process in all corn byproducts except corn starch [16]. It is notable that the most obvious ZEN and DON contaminations occur during the period of preharvest crops. There is also a possibility that mycotoxins could pollute cereal in postharvest crops [17,18]. When moldy corn or germ is used as raw materials for the production of processing products (such as maize germ oil and maize germ meal), pigments, mycotoxins, etc., produced by mold may migrate into the processing products, which may lead the poor qualities of the processing products. Thus, moldy corn or germ may lead to a decrease in the economic value of processing products, as well as edible safety for humans and animals. Nowadays, large quantities of maize germ obtained from corn deep processing is usually sold to maize germ oil production enterprises. Maize germ is usually stored for a period of time before processing. However, there are few reports showing the influence of storage with different condition (initial moisture content, temperature and relative humidity) on the qualities and mycotoxins contaminated levels of maize germ, as well as the corresponding maize germ oil and maize germ meal.

Therefore, this study is aimed to investigate the influence of moisture content and humidity on the qualities and ZEN and DON levels of maize germ and its maize germ oil and meal during maize germ storage. The quality indices of maize germ (moisture content and crude fat content) and maize germ oil (color, acid value and peroxide value) were analyzed. Meanwhile, mycotoxin contamination levels (ZEN and DON) of maize germ and maize germ oil—as well as maize germ meal—were determined. This study would be helpful for the maize-germ processing industry to build reasonable storage conditions of maize germ for reducing the adverse effect of ZEN and DON on maize germ and its processing products.

## 2. Results and Discussion

### 2.1. Quality Changes of Maize Germ During Storage

**Figure 2:**
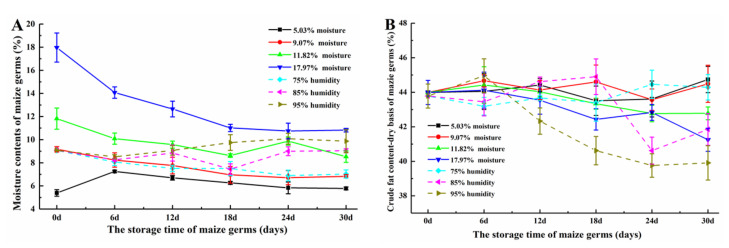
(**A**) Moisture contents and (**B**) crude fat contents of maize germ during storage.

Maize germ with different initial moisture contents (5.03, 9.07, 11.82 and 17.97%) was stored at the situation of 25 °C (temperature)/45% (humidity) for 30 days, while other maize germ with 9.07% initial moisture content was stored at the conditions of 25 °C and different humidity (75, 85 and 95%) for 30 days. The appearance changes of the maize germ were photographed (Figures S1 and S2). The changes of moisture contents and crude fat contents of these stored maize germ samples are shown in Figure 2A,B.

Moisture content and crude fat content of maize germ are two important indexes in reflecting the quality of maize germ. As shown in Figure 2A, the moisture content of germ samples with initial moisture contents of 9.07, 11.82 and 17.97%, respectively, showed similar changing trends during storage at 25 °C and relative humidity of 45%. Their moisture contents all tended to decline over time, which reduced to 6.83 (*p <* 0.05), 8.53 (*p* < 0.05) and 10.82% (*p* < 0.05), respectively. The moisture content of the sample with 5.03% initial moisture content remained almost the same (*p* > 0.05) during storage. The probable reason for this phenomenon may be related to the storage containers and humidity. The raw maize germ materials were stored in open plastic bottle containers and kept under low relative humidity. The germ in the upper position of container was easily desorbed of water, which made the samples with higher moisture contents lose water. Also, it was noticed that the moldy germ was mainly found in the middle and bottom part of the container. As the germ was stored at different humidity, the moisture contents of maize germ showed different changing trends. When the humidity was kept at 75%, the moisture content of germ reduced from 9.07 to 7.03% (*p* < 0.05). However, the germ kept at 85 and 95% humidity exhibited the high final moisture content at 9.04 (*p* > 0.05) and 9.87% (*p* > 0.05), respectively. In the industrial production and utilization of corn germ, the moisture content of corn germ is usually determined according to the requirement of processing technology. Therefore, the moisture content of corn germ is various aiming for different applications, which could range from 3 to 13% [19,20,21,22].

As shown in Figure 2B, the crude fat content of maize germ with initial lower moisture contents (5.03 and 9.07%) showed small changes (fluctuating around 44%, *p* > 0.05) during storage. Meanwhile, the crude fat contents of germ with higher moisture contents (11.82 and 17.97%) was gradually declined during the storage, which decreased to 42.78% (*p* > 0.05) and 41.25% (*p* < 0.05), respectively. Germ (initial moisture content, 9.07%) kept at a lower relative humidity (75%) condition showed similar crude fat content during the storage. The crude fat contents of germ kept at higher relative humidity (85 and 95%) conditions declined by 1.93 and 3.87%, respectively, which was finally decreased to the contents of 41.85 (*p* < 0.05) and 39.91% (*p* < 0.05), respectively. It was speculated that maize germ was prone to occur with moldy situation under the condition of relative high humidity and the crude fat content of germ would decline, resulting in the economic losses. It was seen that germ with lower initial moisture contents (5.03 and 9.07%) as well as kept in lower relative humidity (45 and 75%) did not show mold pollution or had slight moldy kernels during the entire 30-day storage period. However, germ with higher moisture contents (11.82 and 17.97%) or kept in relative higher humidity (85 and 95%) occurred with severe moldy phenomenon at 18th day. The growth and reproduction of mold could resolve a certain amount of fat, which produce substances such as free fatty acids and also lead the decrease of the crude fat content of maize germ [23]. Although the crude fat is decomposed to a certain extent, corn germ still has high fat content. Corn germ is rich in oil and the oil contents range from 35 to 56% [22]. The effect of different corn degerming and fermentation treatments on the germ yields and oil contents was studied; the results showed that the oil contents in germ ranged from 33.50 to 39.16% [24].

### 2.2. Quality Changes of Maize Germ Oils During Storage

**Figure 3:**
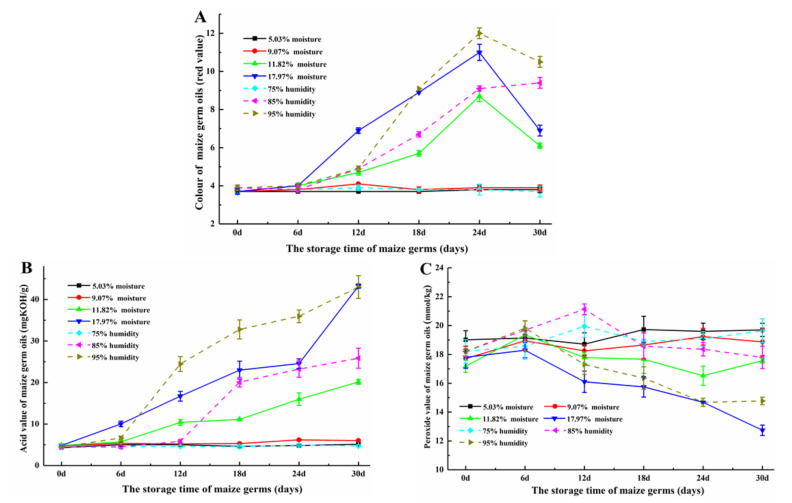
(**A**) colors, (**B**) acid values and (**C**) peroxide values of maize germ oils extracted from germ from different storage stages.

Maize germ taken from different storage periods (0, 6, 12, 18, 24 and 30 days) was used for the solvent extraction to obtain maize germ oils. The quality indices of maize germ oils—including color, acid value and peroxide value—were analyzed (Figure 3A–C). As shown in Figure 3A, the colors of maize germ oils extracted from the germ from different storage periods and with lower initial moisture contents (5.03 and 9.07%) showed no significant differences (*p* > 0.05). On the other hand, the stored germ with a higher initial moisture content (11.82 and 17.92%) could produce deep color in oils, and the red values of these corn germ oils, respectively reached 8.7 (*p* < 0.05) and 11.0 (*p* < 0.05) (yellow value was set as 30) over 24 days of storage. Therefore, there may be a positive correlation between the color of the maize germ oil and the degree of germ moldiness. This is to say that germ with lower initial moisture content exhibited no obvious mold changes and the corresponding oils extracted from them showed light color, while the germ with higher initial moisture content occurred with moldy damage and the corresponding oils showed deep color. In comparing the colors of corn germ oils obtained from the germ stored in different humidity (75, 85 and 95%), it was found that the higher the humidity during germ storage, the higher the degree of moldiness—resulting in a darker color of the corresponding germ oil. Interesting, the red values of corn germ oils (including these oils extracted from the germ with higher initial moisture contents (11.82 and 17.92%) or the germ stored at higher relativity humidity (95%)) appeared slight declined at the end of storage (30 d), which may have a relationship with the growth and reproduction of different mold species at the later period of storage.

The acidity of the germ oils can also change dramatically during storage (Figure 3B). It was seen that the acid values of oils derived from germ with lower initial moisture contents (5.03 and 9.07%) or stored at lower relative humidity (75%) condition stayed in a similar value during the storage (*p* > 0.05), while these oils from germ with higher initial moisture contents (11.82 and 17.92%) or stored at higher relative humidity (85 and 95%) conditions showed the 4–9 times fold (*p* < 0.05) increase of acid values (20.14, 43.22, 25.86 and 42.98 mg KOH/g, respectively). It was suspected that an improper storage condition for maize germ can lead the deterioration of germ oil quality. On one hand, under the action of lipase, the lipid in maize germ was hydrolyzed, leading the production of free fatty acids [25]. On the other hand, the growth of mold may use and break lipids down—also leading to produce free fatty acids. The storage condition with high humidity and moisture content was beneficial for the growth of mold. It could increase the breathing intensity of mold and then produce more heat and finally improve the stored temperature of germ. This situation was more conducive to increasing lipase activity and mold growth rate. Finally, more lipid was decomposed, leading to the production of more free fatty acids. Therefore, it was necessary to control the moisture content of germ and the storage humidity to obtain high quality of maize germ oil. Additionally, the peroxide values of maize germ oils are shown in Figure 3C. It was found that the peroxide values of oils obtained from germ with initial moisture contents (5.03, 9.07 and 11.82%) or stored at relative humidity (75 and 85%) stayed at a similar level (*p* > 0.05). Those oils from germ with 17.92% of initial moisture content or stored at 95% of relative humidity showed a decline of peroxide values (*p* < 0.05). A possible reason for the phenomenon may be related to the blocking effect of water on the lipid oxidation [26]. The quality of corn germ oil is affected by the method of oil extraction, corn germ quality, etc. The effect of moisture and heat treatment of corn germ on oil quality was studied, results showing that increasing the moisture contents of the corn germ from 8 to 25% before oil extraction increased the acid values (3.02–4.01 mg KOH/g), peroxide values (1.04–2.10 mmol/kg) and the red values (7.3–8.7) [27]. An efficient and eco-friendly extraction of corn germ oil was adopted. The obtained crude germ oil showed the acid value with 0.77 mg KOH/g and peroxide value with 10.16 mmol/kg [22]. Another study showed the corn germ oil with a high acid value (10.7 mg KOH/g), which was extracted by Soxhlet extraction using hexane [28].

### 2.3. ZEN and DON Levels in Stored Maize Germ and Its Processing Products

#### 2.3.1. ZEN and DON Levels in Stored Maize Germ

**Figure 4:**
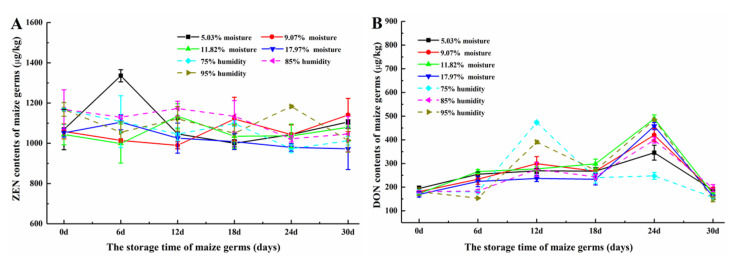
(**A**) ZEN contents and (**B**) DON contents of maize germ during the storage.

Figure 4A shows the ZEN contents of the maize germ from different storage period. The ZEN contents of all maize germ with different storage condition presented little changes during the storage (*p* > 0.05). The ZEN levels of germ with different initial moisture contents and stored under different humidity ranged from 972.44–1335.58 μg/kg and 973.32–1183.16 μg/kg, respectively. However, the average contaminated level of ZEN was 1059.91 and 1092.56 μg/kg, respectively. The DON contents of all maize germ are shown in Figure 4B. With different initial moisture contents (5.03, 9.07, 11.82 and 17.97%), the DON levels of germ remained almost the same during the first 18 days of storage (*p* > 0.05). Then, the DON contents of these four samples, respectively increased to 345.6, 420.1, 489.5 and 457.9 μg/kg at 24th day storage. However, the DON contents of these four samples, respectively declined to 187.2, 180.4, 171.6 and 160.9 μg/kg (all *p*-values of these contents were greater than 0.05 when compared with that of 0th day storage) at 30th day storage. When maize germ was stored in different humidity (75, 85 and 95%), the DON levels of maize germ showed the similar change trends (*p* > 0.05). The DON contents increased at both the 12th day (473.3, 275.3 and 390.4 μg/kg) and 24th day (247.8, 398.7 and 484.7 μg/kg) and then declined to 157.1, 195.8 and 148.3 μg/kg at the 30th day.

ZEN and DON are secondary metabolites of food spoilage fungi mainly produced by *Fusarium*. ZEN is commonly produced by *Fusarium roseum* and *Fusarium moniliforme*, and DON is produced by *Fusarium graminearum* and *Fusarium culmorum*. During the later storage, quantities of turquoise molds as well as part yellow and black molds grew on the corn germ. As reported, *Fusarium* toxin could occurred in all stored corn in Nigeria and the toxin concentrations showed the range of 11–479 μg/kg [29]. The moisture content of the stored maize could also influence the activities of insect pests, which may also be relationship with mycotoxin levels [17,30]. For in-field contaminated cereals, a very significant correlation of DON with regions and meteorological factors (air temperature, precipitation, relative humidity) has been reported [31]. In corn, ZEN and DON may cooccur in preharvest and postharvest and could also increase during storage [32]. In actual processing and transportation, the mycotoxins contamination was a high risk for maize and its byproducts because of the high moisture content and high temperature caused by rain, humidity, etc. [1]. Therefore, in order to control the quality of stored maize germ, the transportation and storage environment of corn germ should be control and ZEN and DON contamination should be monitored.

#### 2.3.2. ZEN and DON Levels in Maize Germ Meals and Maize Germ Oils

**Figure 5:**
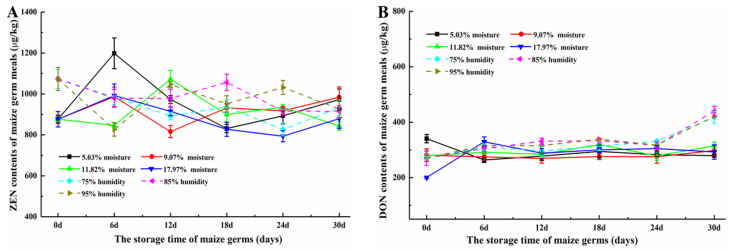
(**A**) ZEN contents and (**B**) DON contents of maize germ meals produced by maize germ from different storage stages. ZEN and DON contents of maize germ meals from all storage stage showed no significant difference (*p* < 0.05), respectively.

**Figure 6:**
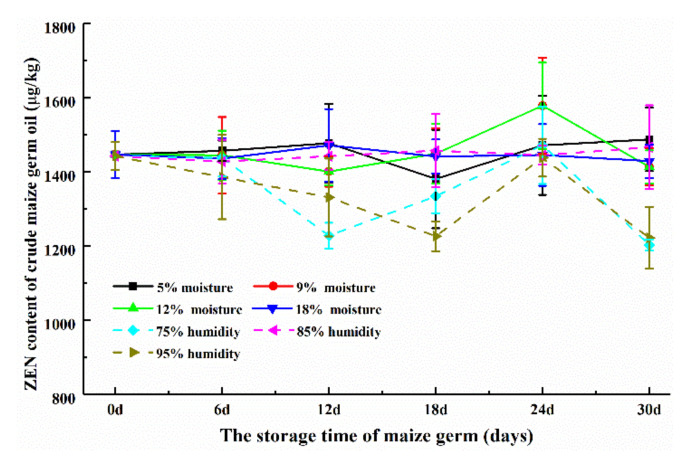
ZEN contents of maize germ oils extracted from the germ from different storage stages. The ZEN contents of maize germ oils from all storage stages showed no significant difference (*p* < 0.05).

Maize germ with different initial moisture contents and stored under different humidity were further processed to obtain maize germ meals and maize germ oils. The contents of ZEN and DON contaminations in those processing products are shown in Figure 5 and Figure 6. As shown in Figure 5A, ZEN contents of meals obtained from germ with different initial moisture contents and stored under different humidity ranged from 793.28 to 1198.73 μg/kg and from 829.41 to 1073.28 μg/kg, respectively. The average ZEN content was 917.27 and 967.92 μg/kg, respectively. Meanwhile, the DON contents of meals ranged from 200.2 to 329.5 μg/kg and from 270.6 to 339.6 μg/kg, respectively. The average DON content was 287.37 and 311.4 μg/kg, respectively (Figure 5B). Though the DON levels of these germ and meals were lower than the maximum limit based on Chinese government standard (1000 μg/kg) [33], the ZEN contents of these germ and meals were all higher than the maximum limit (500 μg/kg) [34], which implied that ZEN pollution was a potential hazard in corn germ and its meal.

As shown in Figure 6, the ZEN contents of maize germ oils extracted from germ with different initial moisture contents and stored at different humidity remained at a similar level and the ZEN contents of these samples showed the range of 1381.08–1578.63 μg/kg and 1203.32–1473.07 μg/kg, respectively. The average ZEN content was 1441.40 and 1380.63 μg/kg, respectively, which were all higher than the maximum limit (400 μg/kg) of corn germ oil [12]. However, the DON contents of all these oil samples were lower than the detection limit of HPLC method (10 μg/kg). The possible reason was that the DON showing low contents in germ (153.5–489.5 μg/kg) mostly transferred into the germ meals at the similar DON levels to germ (240.2–439.6 μg/kg), as DON is much easier to dissolve in the high polarity solvents such as water and acetonitrile than in the low polarity solvents such as oil and hexane [3]. Thirty crude corn oils and 40 refined corn oils were analyzed of DON. Only 20% of crude corn oils and 12.5% of refined corn oils were found of DON with the range of 67.5–340.5 μg/kg and 57.1–207.5 μg/kg, respectively [3]. Vegetable oils has a low incidence of DON, which may be related to the high water-solubility of DON. However, 25% of maize germ and 32% of corn oil samples were positive for ZEN, which may be related to its lipophilic nature [6,35].

#### 2.3.3. Distribution of ZEN and DON in Maize Germ and Its Processing Products

As shown in Figure 4A, the contents of ZEN in all maize germ samples in this study showed the range of 973.32–1335.68 μg/kg. Meanwhile, the contents of ZEN in all germ oils (Figure 5A) and all meals (Figure 6) ranged from 1203.32 to 1578.63 μg/kg and from 793.28 to 1198.73 μg/kg, respectively. The average content of ZEN in maize germ, germ oils and meals were 1074.46, 1421.82 and 938.98 μg/kg, respectively. Figure 4B and Figure 5B show the contents of DON in maize germ and meals, ranging from 148.3 to 489.5 μg/kg and from 200.0 to 439.6 μg/kg, respectively. The average content of DON was 258.22 and 305.04 μg/kg, respectively. While, DON was not found in maize germ oils (Table 1). The contents of ZEN in germ oils were 1.18–1.24 times higher than the contents of ZEN in germ. While the ZEN contents in meals were about 82–90% of that in germ. Vegetable oils do not retain polar mycotoxins, whereas ZEN with less polar can accumulate in vegetable oils [6,36]. It was inferred that the ZEN was inclined to be transferred into oil, while DON was inclined to be transferred into meal during the oil extraction from maize germ, implying that the maize germ oil could have a high risk of ZEN contamination and the meal could have a high risk of DON contamination [3].

## 3. Conclusions

Maize germ was stored with different initial moisture contents or at different relative humidity for 30 days. The stored maize germ and their corresponding germ oils and meals were analyzed of their qualities, DON and ZEN levels. The maize germ obtained from high initial moistures or humidity condition, and their corresponding germ oils showed great changes in qualities at the end of storage. However, the ZEN and DON contents of these maize germ remained at similar levels during the storage, respectively. At the same time, their germ oils and meals also showed the same situation on the ZEN and DON levels. Therefore, the appearance of maize germ was only used for the estimation of fungal contamination of germ, but was not suitable for reflecting the levels of DON and ZEN in maize germ and its germ oil. For obtaining high quality maize germ as used in this study, it was better to keep the maize germ with low initial moisture content (<9%) and at low relative humidity (<75%).

## 4. Materials and Methods

### 4.1. Materials

Acetonitrile and methanol (HPLC grade) were obtained from Merck (Darmstadt, Germany). ZEN and DON standards were procured from Sigma-Aldrich (Saint Quentin-Fallavier, France). The concentrations of ZEN stock solution and DON stock solution were 0.1 and 0.5 mg/mL, respectively. Then the stock solutions of ZEN and DON were diluted with acetonitrile and methanol, respectively, to obtain the working solutions. Both stock solutions and working solutions were kept at −20 °C until used. Immunoaffinity columns of ZEN and DON were purchased from Welchrom^®^ (Welch, Shanghai, China). All the other reagents used were of analytical grade.

### 4.2. Storage of Maize Germ

The moisture content of raw maize germ was adjusted into 5.03, 9.07, 11.82 and 17.97%, respectively and named as Samples 1–4. These samples were contained in open container plastic bottles which were then placed in a constant temperature and humidity incubator under the conditions of the temperature at 25 °C and the relative humidity at 45% for 30 days storage. Other samples of 9.07% moisture content were used and divided into 3 groups which were kept in the open plastic bottle containers under the conditions of 25 °C (temperature)/75% (humidity), 25 °C/85% and 25 °C/95% for 30 d, respectively. These three samples were named as Samples 5–7. Each of these 7 samples used for the storage weighed 1200 g. The dispersity and moldy degree of maize germ was observed during the period. After mixed thoroughly, 200 g maize germ was sampled from each group at 0, 6, 12, 18, 24 and 30 days. Then the moisture contents and crude fat contents of maize germ were determined according to the requirements of Chinese government standards.

### 4.3. Quality Parameters of Stored Maize Germ

#### 4.3.1. Moisture Content

The determination of moisture content referring to GB5009.3-2016 [37] is briefly described below. The clean aluminum weighing bottle was placed in a drying cabinet at 101–105 °C and heated for one hour. Then, the weighing bottle was placed in a desiccator for cooling. The above operation was repeated until the mass change of the weighing bottle did not exceed 2 mg. About 5 g of sample was grounded and weighted in the weighing bottle and then placed in the drying cabinet at 101–105 °C for two hours. After the cooling in a desiccator, the sample was weighed. Then, the sample was heated repeatedly until the mass change of the weighing bottle did not exceed 2 mg. The moisture content of sample was calculated according to the value difference.

#### 4.3.2. Crude Fat Content

The conventional Soxhlet extraction (SE) was selected for the determination of crude fat content according to previous report [38]. The sample was extracted for 8 h using diethyl ether at 60 °C. The small amount of diethyl ether remaining in the receiving bottle was removed in an drying cabinet at 101–105 °C for one hour.

### 4.4. Preparation of Maize Germ Oil and Meal

The stored maize germ sample was crushed and sieved though a 20-mesh sieve. Smashed maize germ and n-hexane were mixed in a sealed container. The container was put into a water bath (50 °C) and stirred for 8 h. Then, the mixture was filtered through a Buchner funnel under vacuum and the filtrate was collected. The maize germ oil was gained by removing the solvent from the filtrate by the vacuum rotary evaporator. The maize germ meal was obtained after removing n-hexane from the filter residue. Then, the quality parameters (color, acid value and peroxide value) of maize germ oil were detected.

### 4.5. Quality Parameters of Maize Germ Oil

#### 4.5.1. Color

The color of maize germ oil was detected according to GB22460-2008 [39]. The mothed was briefly described as below. A certain amount of maize germ oil was injected into the 25.4 mm colorimetric tank. The liquid level of oil was about 5 mm away from the upper opening of the colorimetric tank. Then, the colorimetric tank was placed into Lovibond Tintometer (Perkone Technology Co., Ltd., Hangzhou, China). In order to compare the color difference of oil sample, the yellow value was set as 30. The red value was adjusted until the color of the shade guide was approximately same as the color of oil sample.

#### 4.5.2. Acid Value

The determination of acid value in maize germ oil was carried out using the national standard GB/T 5009.229-2016 [40]. The main operational procedure was conducted as follows: About 2 g of oil sample was dissolved in 25 mL mixed solvent (ether:isopropanol = 1:1, *v*/*v*). The mixture was shaken well, and three drops of phenolphthalein indicator were added to the mixed solution and then titrated with potassium hydroxide standard solution (0.1000 mol/L). The acid value was subsequently calculated.

#### 4.5.3. Peroxide Value

The peroxide value of maize germ oil was determined based on the method as described before [41]. Briefly, about 2 g maize germ oil was added in an iodine flask and then 30 mL mixed solvent (chloroform:acetic acid = 3:2, *v*/*v*) and 1 mL saturated potassium iodide solution were added orderly. The mixed solution was shaken and placed in the dark for reaction (5 min). After the reaction, 100 mL of distilled water and three drops of starch indicator were added and followed by titrating with the Na_2_S_2_O_3_-solution.

### 4.6. Mycotoxin Levels in Maize Germ and Its Processing Products

#### 4.6.1. ZEN Levels of Maize Germ and Its Processing Products

The ZEN levels of the samples were detected by the method as described before with slight modification [9,42]. Briefly, a 5-g sample was mixed with 25 mL acetonitrile–deionized water (8:2, *v*/*v*) as well as 1.0 g sodium chloride. Then the mixture was homogenized by a homogenizer (FM200, FLUKO^®^, Shanghai, China) and centrifuged at 7000× *g* for 3 min. After filtrated with glass-fiber filter paper, the filtrate was slowly passed through a ZEN immunoaffinity column and followed by PBS solution, deionized water and flushing air. The methanol was added into the column to elution the sample. The solution was collected, dried and then dissolved with acetonitrile, and finally filtered through a 0.22-μm syringe for the analysis of ZEN by HPLC method.

The HPLC (Waters 2695, Waters Corp., Milford, MA, USA) equipped with Waters 2495 fluorescence detector (FI) was used to analyze sample. The sample was separated by a SunFire^®^ C18 column (4.6 × 150 mm, 5 μm, Waters, Wexford, Ireland), which was located in a column oven set at 35 °C. The eluent was consisted of acetonitrile and water (55:45, *v*/*v*) with a speed rate of 1.0 mL/min. The injection volume was 20 μL. The excitation wavelength was set at 278 nm and the emission wavelength was set at 462 nm.

#### 4.6.2. DON Levels of Maize Germ and Its Processing Products

The DON levels of the samples were determined by the following method described previously with some modifications [3,8,43]. In brief, a 12.5-g sample was mixed with 100 mL deionized water and 5 g polyethylene glycol 8000. After sufficient homogenization, the mixture was centrifuged and filtrated with glass-fiber filter paper. Then, the filtrate was passed through a DON immunoaffinity column at a low speed rate. A certain amount of air was passed through the column to exclude the residual liquid. Subsequently, the column was washed with PBS solution, deionized water and flushing air. Finally, the elution was completed with methanol. The eluent was dried under a gentle stream of N_2_. After dissolving with solution (methanol:deionized water = 20:80, *v/v*), the sample was filtered through a 0.22-μm syringe and used for instrument analysis.

The HPLC equipped with Waters 2489 ultraviolet detector (UV) was used for the analysis of DON content. A SunFire^®^ C18 column (4.6 × 250 mm, 5 μm, Waters, Ireland) coupled with a SunFire^®^ guard column (5 μm, Waters, Ireland) was used to separate sample at 35 °C with a flow rate of 0.25-mL/min and a 50-μL injection volume. The elution process was performed with methanol and water (20:80, *v*/*v*) and the detection wavelength was set at 218 nm.

### 4.7. Statistical Analysis

The statistical analysis was performed using the Waters-Empower 3 software (Waters Crop., Milford, MA, USA) and SPSS 22.0 (IBM Corporation, Armonk, New York, USA). Figures were drawn with Origin 8 (Origin Lab, Northampton, MA, USA). All experiments were carried out in triplicate, and data were expressed as mean ± standard deviation. The mean values were compared with one-way ANOVA, followed by Dunnett’s test and the *p* < 0.05 stood for significant difference.

## Figures and Tables

**Table 1:** DON contents of maize germ oils extracted from germ from different storage stages.

Days No.	0 d	6 d	12 d	18 d	24 d	30 d
No. 1	ND	ND	ND	ND	ND	ND
No. 2	ND	ND	ND	ND	ND	ND
No. 3	ND	ND	ND	ND	ND	ND
No. 4	ND	ND	ND	ND	ND	ND
No. 5	ND	ND	ND	ND	ND	ND
No. 6	ND	ND	ND	ND	ND	ND
No. 7	ND	ND	ND	ND	ND	ND

No. 1–No. 4—moisture content of 5, 9, 12 and 18%, respectively. No. 5–No. 7—environmental humidity of 75, 85 and 95%, respectively. “ND”—not detected.

## Supplementary Materials

The following are available online at https://www.mdpi.com/2072-6651/12/9/535/s1, Figure S1: The appearances of maize germ during the 30-day storage with different initial moisture contents, Figure S2: The appearances of maize germ during the 30-day storage at different relatively humidity.
