# Supplementary material for: Effect of Stored Humidity and Initial Moisture Content on the Qualities and Mycotoxin Levels of Maize Germ and Its Processing Products
Source: Toxins (Basel). 2020 Aug 20;12(9):535. doi: 10.3390/toxins12090535 (PMC7551338; doi:10.3390/toxins12090535)
Supplement: Supplementary file 1 [file toxins-12-00535-s001.pdf]

Supplementary Materials: Effect of Stored Humidity and Initial Moisture Content on the Qualities and Mycotoxin Levels of Maize Germ and Its Processing Products

Yun-qi Wen, Li-li Xu, Chang-hu Xue and Xiao-ming Jiang

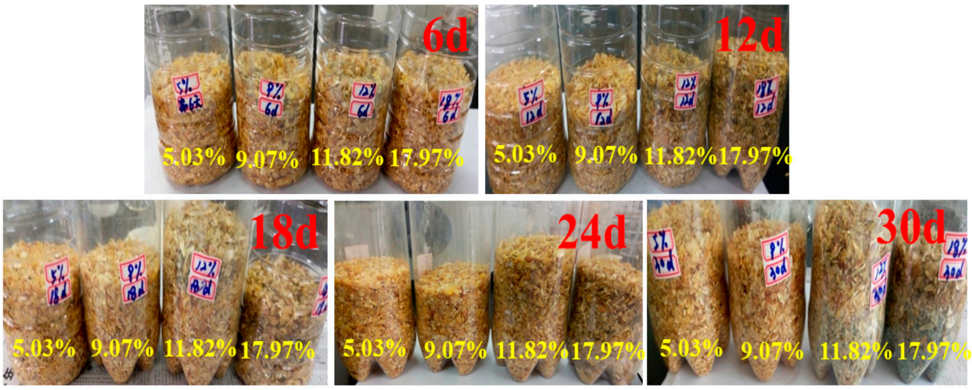

Figure S1. the appearances of maize germs during the 30 days storage with different initial moisture contents.

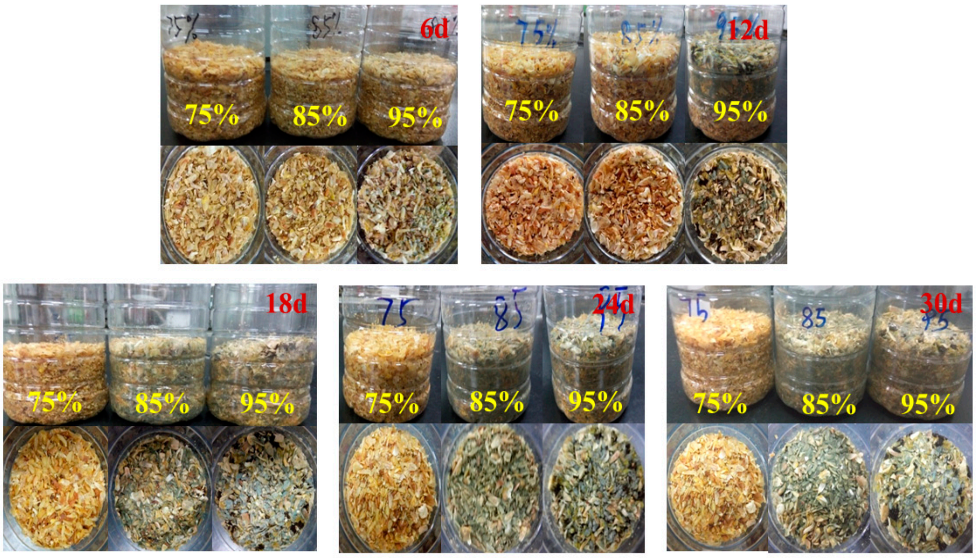

Figure 2S. the appearances of maize germs during the 30 days storage at different relatively humidity.
